# Shared and Unique Neural Codes for Biological Motion Perception in Humans and Macaque Monkeys

**DOI:** 10.1002/advs.202411562

**Published:** 2025-03-16

**Authors:** Yuhui Cheng, Yumeng Xin, Xiqian Lu, Tianshu Yang, Xiaohan Ma, Xiangyong Yuan, Ning Liu, Yi Jiang

**Affiliations:** ^1^ State Key Laboratory of Cognitive Science and Mental Health, Institute of Psychology Chinese Academy of Sciences Beijing 100101 China; ^2^ University of Chinese Academy of Sciences Beijing 100049 China; ^3^ State Key Laboratory of Cognitive Science and Mental Health, Institute of Biophysics Chinese Academy of Sciences Beijing 100101 China; ^4^ School of Psychology Nanjing Normal University Nanjing 210097 China; ^5^ Department of Radiology, Renji Hospital, School of Medicine Shanghai Jiao Tong University Shanghai 200025 China

**Keywords:** biological motion, cross‐species comparison, evolution, fMRI, non‐human primates

## Abstract

Throughout evolution, living organisms have honed the ability to swiftly recognize biological motion (BM) across species. However, how the brain processes within‐ and cross‐species BM, and the evolutionary progression of these processes, remain unclear. To investigate these questions, the current study examined brain activity in the lateral temporal areas of humans and monkeys as they passively observed upright and inverted human and macaque BM stimuli. In humans, the middle temporal area (hMT+) responded to both human and macaque BM stimuli, while the right posterior superior temporal sulcus (hpSTS) exhibited selective responses to human BM stimuli. This selectivity is evidenced by an increased feedforward connection from hMT+ to hpSTS during the processing of human BM stimuli. In monkeys, the MT region processed BM stimuli from both species, but no subregion in the STS anterior to MT is specific to conspecific BM stimuli. A comparison of these findings suggests that upstream brain regions (i.e., MT) may retain homologous functions across species, while downstream brain regions (i.e., STS) may have undergone differentiation and specialization throughout evolution. These results provide insights into the commonalities and differences in the specialized visual pathway engaged in processing within‐ and cross‐species BMs, as well as their functional divergence during evolution.

## Graphical abstract

Cross‐species fMRI studies reveal distinct neural mechanisms for biological motion (BM) processing. In humans, the posterior superior temporal sulcus (hpSTS) selectively responds to conspecific BM, while monkeys process BM from both species in the middle temporal area (MT). This suggests conserved MT functions but specialized STS evolution, highlighting species‐specific adaptations in visual pathways for BM recognition.

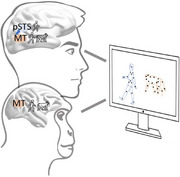

## 1 Introduction

Biological motion (BM) refers to the recognizable movement patterns generated by the major joints of living creatures,^[^ ^1^, ^2^ ^]^ conveying not only their movement status but also their social intention.^[^ ^3^, ^4^ ^]^ Therefore, the efficient recognition of BM signals is a cornerstone ability for survival adaptation and social development. On the one hand, animals may develop a species‐general ability to identify the movements of organisms from various species for successful daily‐life activities (e.g., fight‐or‐flight) in a complex external environment.^[^ ^5^, ^6^, ^7^, ^8^ ^]^ This intrinsic ability emerges early in life and may be inheritable,^[^ ^9^, ^10^, ^11^ ^]^ suggesting that it is evolutionarily ancient.^[^ ^7^, ^12^, ^13^ ^]^ On the other hand, animals may be proficiently tuned to the movements of their conspecies, since conspecific BM signals contain a wealth of socially relevant information on which social animals can rely to interact effectively with each other and engage in collaborative group activities.^[^ ^14^ ^]^ Plenty of studies have reported that humans are highly sensitive to conspecific information,^[^ ^8^, ^15^, ^16^, ^17^ ^]^ confirming the evolution of such a species‐specific mechanism.

Hitherto, although the mechanisms underlying BM perception can be characterized as species‐general and species‐specific, it has not been well understood yet whether there are corresponding neural substrates that respectively underscore the species‐general and species‐specific BM processing, and how these neural mechanisms develop and evolve across subjects with the same ancestor. The present study addressed these issues using a cross‐species design, in which humans and non‐human primates (i.e., macaque monkeys) who share a common ancestor and have the closest evolutionary relationships, are engaged in a parallel study to investigate their brain activities associated with within‐ and cross‐species BM perception.

For humans, research has demonstrated that processing BM information activates multiple regions in the superior temporal sulcus (STS), middle temporal cortex (MT), fusiform gyrus (FFG), portions of the frontal and parietal cortex,^[^ ^18^, ^19^, ^20^ ^]^ as well as the left cerebellum and some subcortical brain regions.^[^ ^21^, ^22^, ^23^, ^24^ ^]^ Among them, the most consistently involved are the human MT (hMT+) and the human posterior STS (hpSTS).^[^ ^19^ ^]^ Similar brain regions selectively sensitive to BM stimuli are found in the temporal cortex of monkeys, including the MT complex, anterior regions of the fundus of the STS (e.g., the lower superior temporal region(LST), and the dorsal bank of the STS (e.g., the TEO region).^[^ ^25^, ^26^, ^27^, ^28^ ^]^ Recently, it has been proposed that social dynamic information (including BM and moving faces) in humans and monkeys mainly transmits in a third visual pathway from the primary visual cortex via MT into STS.^[^ ^29^ ^]^ The brain regions in the upstream and downstream of this pathway (e.g., MT and STS) are selectively tuned to hierarchical aspects of the visual signals, from simple motion patterns ^[^ ^30^ ^]^ to action intentions.^[^ ^27^, ^31^ ^]^ Despite monkeys sharing an overall similarity with the human visual system,^[^ ^32^ ^]^ there are some disparities in terms of motion perception. In particular, the homologies between monkey STS and human occipito‐temporal cortex are still unclear.^[^ ^29^ ^]^

Based on the above findings, the current study focused on these two key brain regions (MT and STS) in the third visual pathway, and examined whether within‐ and cross‐species BMs, which primarily differ from each other in social aspects, may activate the two brain areas differently, and whether humans and macaque monkeys process the within‐ and cross‐species BMs in a similar or different manner. We used functional magnetic resonance imaging (fMRI) to measure brain activity in both human and monkey participants as they viewed human and macaque BM stimuli. The brain responses to upright and inverted versions of the BM stimuli were compared to examine the neural correlates of animal movements while minimizing the influence of low‐level visual factors.^[^ ^7^, ^21^, ^33^ ^]^ We applied univariate analysis, multivoxel pattern analysis (MVPA) within and across species on the whole brain and regions of interest (ROIs) in both humans and monkeys, as well as brain connectivity analysis to reveal the respective species‐general and species‐specific neural mechanisms related to BM perception.

## 2 Results

### 2.1 Human MT Generally Processes Cross‐Species BMs, While Human pSTS is Specialized for Processing Human BM

**Figure 1:**
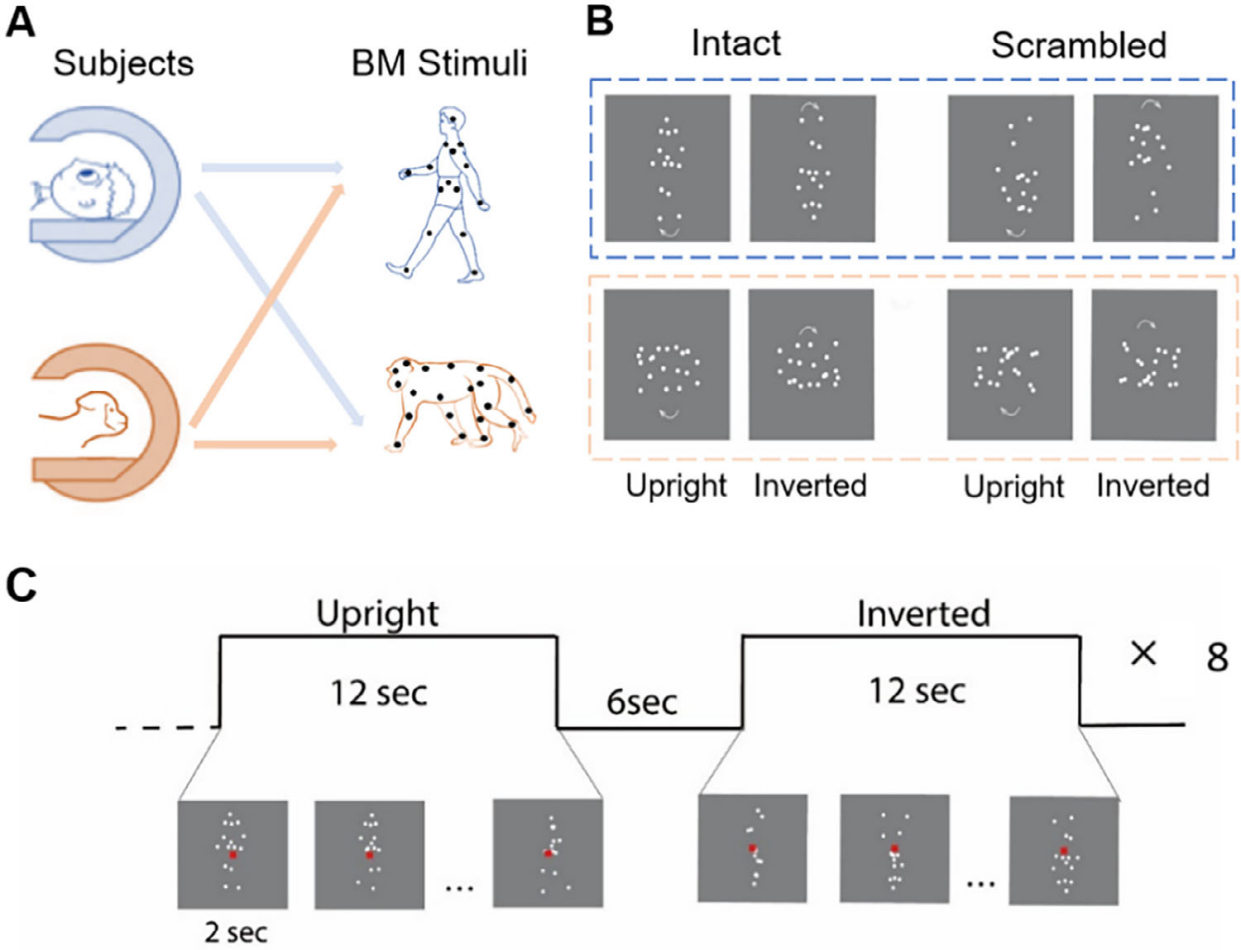
Schematic illustration of experimental design and sample stimuli. A) Human and monkey participants viewed both human and macaque BM stimuli during the fMRI scans. B) Intact human BM scrambled human BM, intact macaque BM, and scrambled macaque BM stimuli were used in the human and monkey fMRI experiments, including their upright and inverted versions. Arrows indicated the motion direction and were not presented in the actual experiments. C) Timeline of a blocked‐design exemplar run for human intact point‐light stimuli in the main task.

During the fMRI scan, human participants viewed the upright and inverted versions of point‐light human or macaque BM (see **Figure** **1**). The participants were asked to perform an orthogonal task involving the detection of color changes in the fixation point, to monitor their sustained attention on the display throughout the scan. The mean accuracy of the participants across all types of BM stimuli was 84.46 ± 22.55%. There were no significant differences in accuracy between the upright and inverted versions of each type of BM stimuli (*ps* > 0.299 for all comparisons), indicating that the participants were able to uniformly focus their attention on both the upright and inverted versions across different types of BM stimuli.

**Figure 2:**
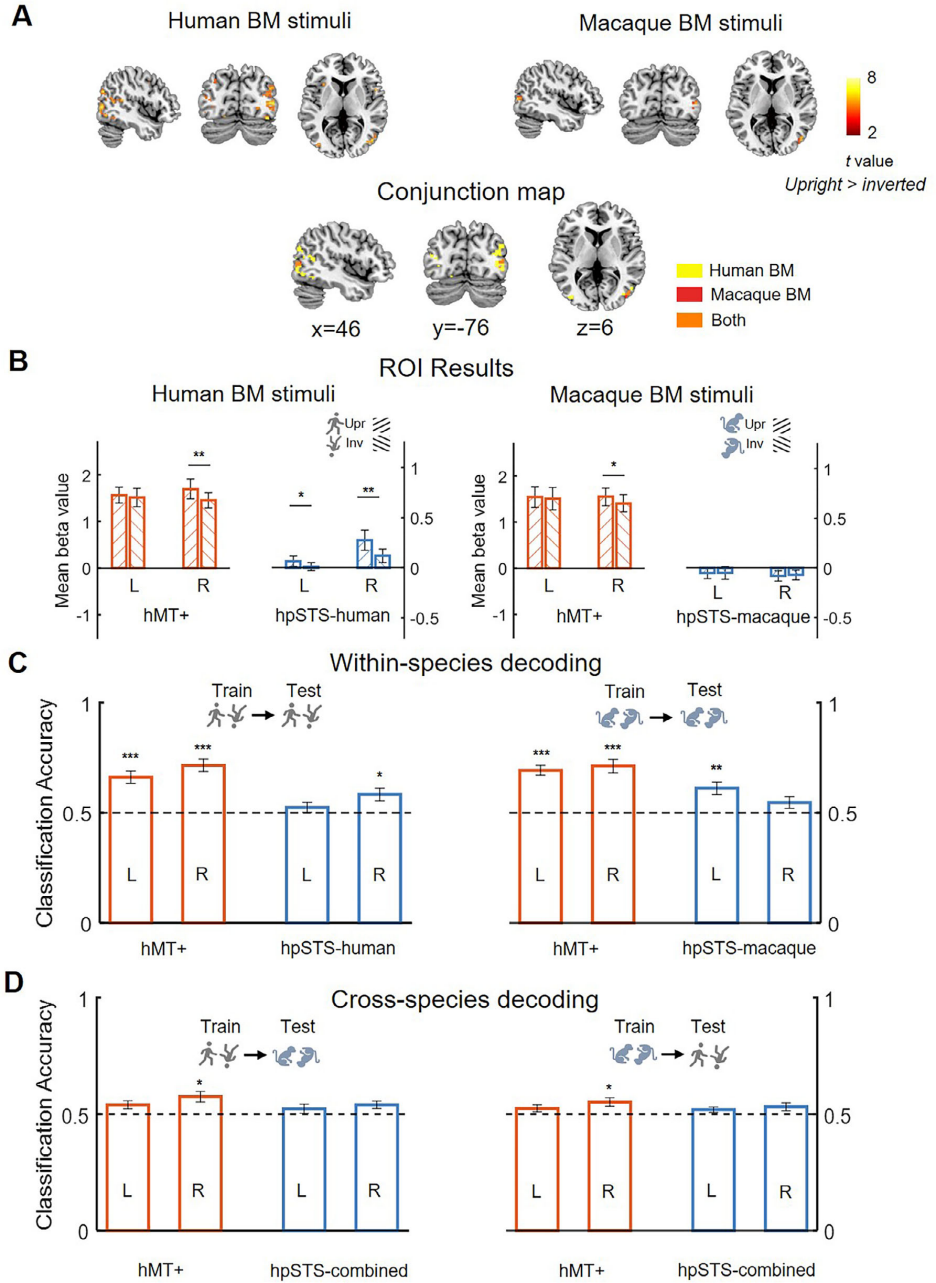
Group‐level fMRI activation maps of human whole‐brain and ROI results. A) Regions depicting areas of activation to the upright versus inverted human and macaque BM stimuli with an FDR‐corrected threshold of *p* < 0.05 at the cluster level and *p* < 0.001 uncorrected at the voxel level (the minimum cluster size > 10 voxels). The color bar indicates *t* values. The common region identified by the conjunction analysis of human BM and macaque BM. B) Mean beta values across participants, in each individually defined left and right hMT+ and hpSTS, in response to upright versus inverted BM, respectively. Error bars denote standard errors of the mean. C,D) The human within‐species C) and cross‐species D) MVPA results of decoding upright and inverted BM stimuli in the hMT+ and hpSTS. The horizontal dashed line represents the chance level (50%). Error bars denote standard errors of the mean. In panel B, the asterisk indicates statistically significant differences between the upright and inverted conditions. In panel C, the asterisk indicates that the classification accuracy is significantly higher than chance level (Bonferroni corrected for 4 comparisons). *p < 0.05, **p < 0.01, ***p < 0.001. The red and blue bars represent hMT+ and hpSTS, respectively. Moreover, a leftward slash indicates the upright condition, whereas a rightward slash indicates the inverted condition.

First, we conducted a whole‐brain analysis to provide an unbiased and exploratory overview of brain activity patterns. Results showed that processing intact human BM stimuli (upright > inverted) elicited increased neural activity encompassing the lateral occipital cortex, left inferior frontal gyrus, right postcentral gyrus, left inferior parietal lobule, and right middle temporal gyrus extending to the posterior superior temporal sulcus. Processing intact macaque BM stimuli only elicited stronger brain activation in the bilateral cuneus and middle occipital gyrus (extending to the middle temporal gyrus; see **Figure** **2**A; also refer to Supplementary results and Table S1, Supporting Information for more details). A conjunction analysis showed that the brain activation for human and macaque BMs overlapped in the right middle temporal (Figure 2A, peak at x = 42, y = ‐76, z = 6, 55 voxels, with a voxelwise FDR‐corrected threshold of *p* < 0.05). Given that the human scrambled BM stimuli did not elicit significant brain activation between upright and inverted contrast in the current univariate analyses (available in Supplementary material Section B), we did not further discuss them in the main text and focused on the results obtained from the intact BM stimuli.

To further examine species‐general and species‐specific BM processing in the third visual pathway, we defined three brain regions using independent localizer tasks (i.e., hMT+, hpSTS‐human, and hpSTS‐macaque). It's worth noting that the hpSTS‐human ROI was used during human BM perception, while the hpSTS‐macaque ROI was used during macaque BM perception (see the Method section, Table S2, and Figure S1, Supporting Information for more details). A 2 (Hemisphere: left versus right) × 2 (Orientation: upright versus inverted) repeated measures analysis of variance (ANOVA) was performed for each type of BM stimuli (e.g., intact human BM). Subsequently, a post‐hoc analysis was conducted to further elucidate the specific differences between upright and inverted conditions. The results are shown in Figure 2B, with detailed statistics in Table S3 (Supporting Information). In brief, we found that the upright human BM, relative to their inverted counterparts, significantly activated the right hMT+ (right: *t*_17_ = 3.56, *p* = 0.002) and bilateral hpSTS‐human ROIs (left: *t*_17_ = 2.63, *p* = 0.017; right: *t*_17_ = 3.89, *p* = 0.001). Similarly, the upright macaque BM, compared with the inverted one, selectively activated the right hMT+ (*t*_17_ = 2.79, *p* = 0.013). Conversely, no significant effect was observed in the hpSTS‐macaque ROI (*F*s < 0.1, *ps* > 0.750). In sum, the ROI results again confirmed the whole‐brain results, showing that the hMT+ is generally dedicated to cross‐species BM perception, whereas the hpSTS, especially in the right hemisphere, is specifically dedicated to same‐species BM perception.

Using MVPA, we further tested whether the brain activation pattern in the hMT+ and hpSTS differentially represents within‐ and cross‐species BM stimuli. First, we trained the classifier to categorize the brain activation patterns (beta values) for the upright versus inverted human/macaque BM stimuli in the predefined hMT+ and hpSTS ROIs, respectively. We then tested the classification accuracy on the leave‐out sample. We found that the classification accuracies in the left and right hMT+ were significantly above the chance level for the BM displays (Figure 2C and Table S4, Supporting Information). This result suggests that the hMT+ ROI has the ability to represent cross‐species BM. But contrary results are found in the hpSTS ROI. The activity pattern in the left and right hpSTS region seemed to separately recognize BM stimuli from the same species (human) and other species (macaque).

To further examine how within‐ and cross‐species BM stimuli are represented in the hMT+ and hpSTS, we trained the classifier to categorize the brain activation patterns of upright and inverted BM stimuli from one species (e.g., human BM) and tested its classification accuracy from the other species (e.g., macaque BM). It was more evident that only the right hMT+ could effectively discriminate the brain activation patterns of upright and inverted BM stimuli irrespective of the species (train human BM and test macaque BM: *t*_17_ = 3.24, *p* = 0.019; train macaque BM and test human BM: *t*_17_ = 2.86, *p* = 0.044; Figure 2C and Table S5, Supporting Information). The MVPA results clearly supported that the hMT+ can generally decode cross‐species BMs. Since the hpSTS‐human/macaque ROIs were identified separately for the human and macaque BM stimuli in the localizer task and did not fully overlap with each other, we defined a new hpSTS‐combined ROI for the cross‐species MVPA to minimize the risk of bias introduced by species‐specific ROI definitions (contrast: human + macaque upright intact BM > human + macaque inverted scrambled BM; please see the methods for more details). No significant decoding performances were found, suggesting that the hpSTS‐combined may not decode cross‐species BMs (Statistics are in the Supplemental Material Section C).

### 2.2 The Feedforward Connectivity from hMT+ to hpSTS is Selectively Modulated by Human BM Perception

Having revealed the species‐general and specific properties of BM processing in the third visual pathway, it is intriguing to explore how the within‐ and cross‐species BM information is transmitted from MT to STS regions using functional connectivity analysis. We first implemented an exploratory psychophysiological interaction (PPI) analysis using the bilateral hMT+ as a search seed. The results showed that the right STS area (x = 64, y = ‐36, z = 6; Voxel‐level: *p* < 0.001 uncorrected; Cluster‐level (FWE): *p* < 0.05 corrected) was co‐activated with the bilateral hMT+ when the human BM stimuli were displayed. Other brain areas functionally connected with the bilateral hMT+ included the left superior frontal gyrus, the bilateral cingulate gyrus, the left anterior cingulate, the left supramarginal gyrus, and the right middle temporal gyrus (see Figure S2 and Table S6, Supporting Information). However, no significant clusters with the predefined *p* value were revealed when the same PPI analysis was performed for the macaque BM stimuli.

**Figure 3:**
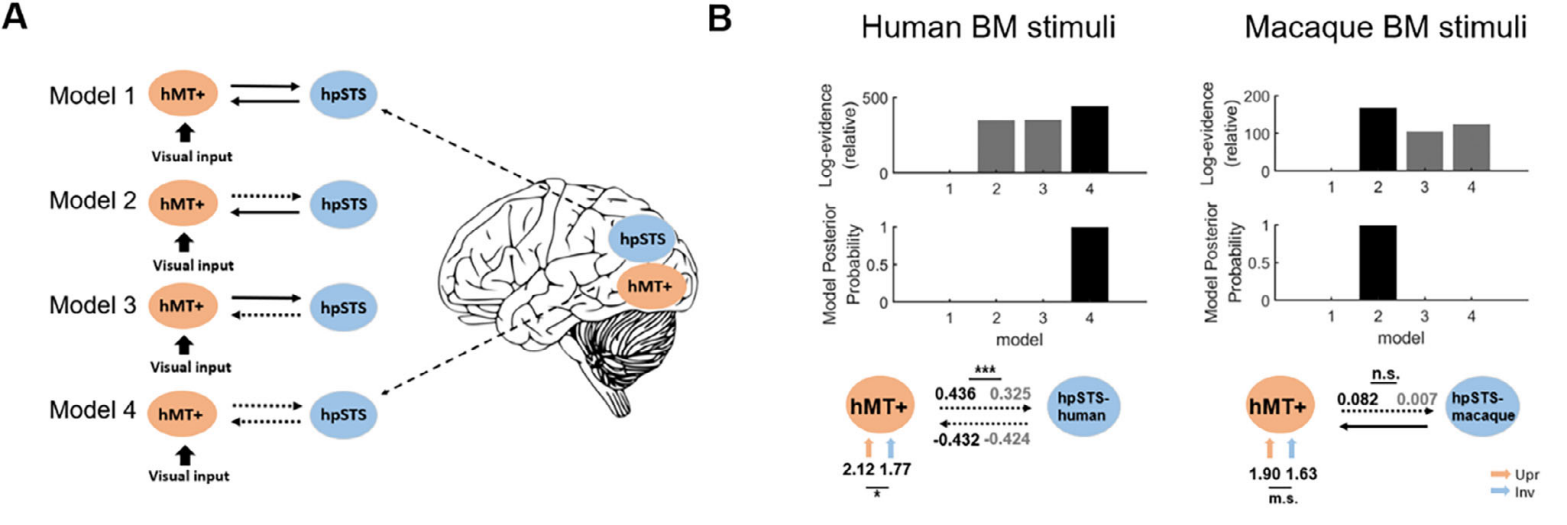
Models and results of DCM. A) The four DCM models between hMT+ and hpSTS are presented from top to bottom, in the following order: “no modulation”, “forward modulation”, “backward modulation”, and “bidirectional modulation”. The solid bold arrows indicate the driving visual inputs. The horizontal black arrows indicate the intrinsic connections between hMT+ and hpSTS. The horizontal dotted arrows indicate the intrinsic connections assumed to be modulated by BM perception. B) The DCM results for human and macaque BM perception (upright versus inverted). The top panels show the log‐evidence and the posterior probability for all models. The bottom panels show the parameters for driving inputs and modulatory effects (expressed in Hz). The black and the gray arrows separately indicate the parameters from the upright and inverted BM. *n.s*., not significant, *m.s*., marginally significant, ∗*p* < 0.05, ∗∗∗*p* < 0.001.

To evaluate the directional functional connectivity between hMT+ and hpSTS, we conducted a dynamic causal modeling (DCM) analysis. In this analysis, we used the hpSTS‐human ROI for human BM stimuli and the hpSTS‐macaque ROI for macaque BM stimuli to ensure the sensitivity of the ROIs to the BM stimuli they preferred (Similar results were obtained when using the hpSTS‐combined ROI; see the Supplemental Material Section C). First, we assumed that the driving visual inputs, including the upright and inverted BM stimuli, enter the system through the hMT+ (C‐matrix), and that the intrinsic connections between hMT+ and hpSTS are all bidirectionally fixed (hMT+ ↔ hpSTS; A‐matrix). We then systematically assessed the connections mediated by BM processing (upright > inverted) across the following four models: 1) no modulation, 2) forward modulation, 3) backward modulation, and 4) bidirectional modulation (**Figure** **3**A). The results indicated that the “bidirectional modulation” had the highest posterior probability for the human BM stimuli, while the “forward modulation” had the highest posterior probability for the macaque BM stimuli (Figure 3B). For the two best models, the driving inputs of the upright BM stimuli were significantly or marginally significantly stronger than those of the inverted ones for both the human (*t*_17_ = 2.12, *p* = 0.047) and macaque BM perception (*t*_17_ = 1.82, *p* = 0.087). However, for the human stimuli only, the modulatory effect of the upright BM was significantly stronger than that of the inverted BM on the forward connection from hMT+ to hpSTS (*t*_17_ = 4.76, *p* < 0.001), but not on the backward connection (*t*_17_ = ‐0.02, *p* = 0.981). For the macaque stimuli, the modulatory effect on the forward connection was not significant (*t*_17_ = 1.25, *p* = 0.229), and no backward connection was found. Taken together, these PPI and DCM results consistently demonstrated that the functional connection between hMT+ and hpSTS, especially the feedforward one, is indispensable for within‐species BM perception. Therefore, it is the within‐species BM, but not the cross‐species BM, information decoded in the hMT+ that is selectively transmitted to the hpSTS.

### 2.3 Monkey MT Regions Generally Respond to Cross‐Species BM

Similar to the procedure for human participants, monkeys passively observed different types of BM stimuli. They were required to maintain fixation on a central square on the screen throughout the entire experimental session and received a liquid reward. Their eye position was tracked to ensure that there was no significant difference between the fixation percentages of the upright and inverted conditions for each type of BM stimuli (*ps* > 0.058). That is, monkeys uniformly focused their attention on both the upright and inverted versions across different types of BM stimuli.

**Figure 4:**
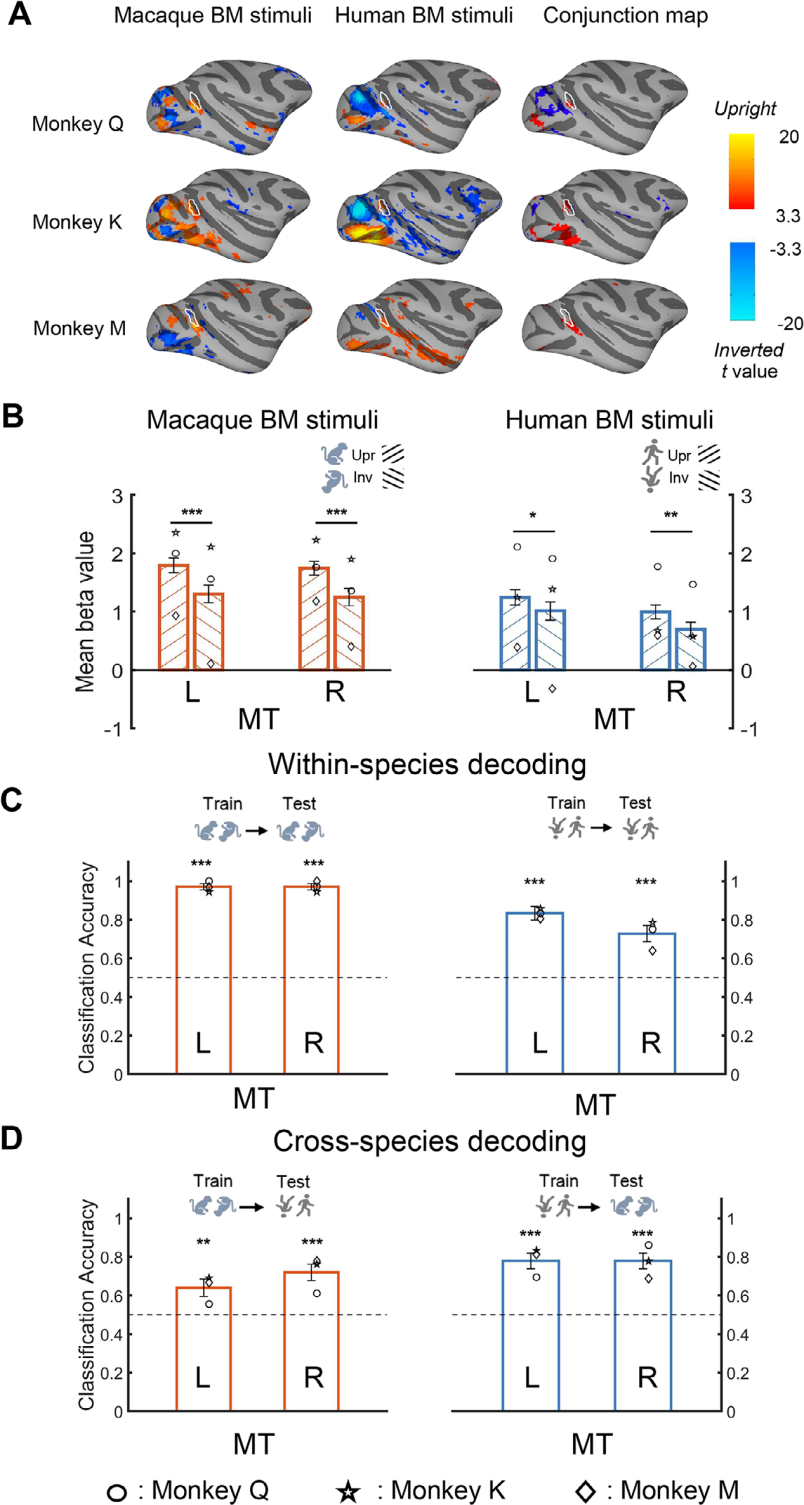
Monkey whole‐brain analyses and ROI results. A) Brain activation maps of three monkeys for the BM stimuli (upright versus inverted), shown on lateral views of the inflated cortex of the right hemisphere. Panels from top to down represent the results of monkeys Q, K, and M, respectively. Panels from left to right show results from the macaque and human BM stimuli (uncorrected *p* < 0.001, FDR corrected *p* < 0.05), and the conjunction maps based on them (FDR corrected *p* < 0.05), respectively. The borders of the MT ROI are encircled by white lines. B) Results of the ROI analysis in the MT. Error bars indicate standard errors. C,D) The monkey within‐species C) and cross‐species D) MVPA results of decoding upright and inverted BM stimuli in the MT. Error bars indicate standard errors. The symbols (circle, star, and diamond) within individual bars represent results from monkeys Q, K, and M, respectively. The black dashed lines indicate the chance level (50%). *p < 0.05; **p < 0.01, ***p < 0.001.

Using univariate whole‐brain analysis with the contrast of upright > inverted BM, we primarily searched for brain activation in the third visual pathway (e.g., MT and STS) that underlies within‐ and cross‐species BM perception in the monkey subjects (**Figure** **4**A). The upright macaque BM stimuli elicited significantly stronger neural activities in the MT, V4t, and V4 areas across all three monkeys than the inverted counterparts did, while the human BM stimuli produced a similar but less pronounced activation in these regions. These results demonstrated that the monkey MT and neighboring brain regions were able to encode both same‐species and cross‐species BMs. Indeed, the conjunction analyses confirmed this result (Figure 4A). However, we did not find any other significant activations in the STS regions anterior to MT with the same contrast across the three monkeys.

Following the analysis protocol of the human experiment, we further examined the above results using ROI analysis (see the Method section along with Figure S4 and Table S8, Supporting Information for details). We replicated the whole‐brain results in the MT ROI by performing a 2 Hemisphere × 2 Orientation Linear Mixed Models (LMMs) with run, session, and monkey as random factors. In short, for the macaque BM stimuli, stronger activation elicited by the upright BM stimuli compared to the inverted ones was found in the bilateral MT ROIs (left: *t*_153_ = 5.75, *p* < 0.001; right: *t*_153_ = 5.80, *p* < 0.001). A similar activation pattern was also found for the human BM stimuli in the bilateral MT ROIs (left: *t*_168_ = 2.26 *p* = 0.025; right: *t*_168_ = 2.89, *p* = 0.004). Note that, in both the human and monkey BM localizers, we found no clusters in the STS area that responded more strongly to the upright intact BM stimuli than the inverted scrambled ones (except those areas close to and overlapped with the MT ROI in the monkey BM localizer, see Figure S4, Supporting Information). Detailed statistics can be found in Table S9 (Supporting Information). In sum, echoing the whole‐brain results, the monkey ROI results indicated the general abilities to process BM from different species in the MT region, while the STS region does not respond to BM perception (upright > inverted) in monkeys, differs from that in humans.

Using within‐species MVPA, we measured whether the upright and inverted BM stimuli from the same species could be discriminated in the MT ROI (Figure 4C). The classification accuracies in the bilateral MT ROIs were significantly higher than the chance level for both the macaque BM and human BM (see Table S10, Supporting Information for statistical values). Furthermore, we performed MVPA across the BM stimuli from different species (Figure 4D and Table S11, Supporting Information). The classification accuracies in the bilateral MT were significantly higher than the chance level, either by training on the human stimuli and testing on the macaque stimuli (left: 77.88 ± 4.07%, right: 77.88 ± 4.07%, *ps* < 0.001), or by training on the macaque stimuli and testing on the human stimuli (left: 64.04 ± 4.49%, right: 71.93 ± 4.21%, *ps* < 0.004). Overall, the monkey MVPA results indicated that the MT ROIs can encode both the within‐species and cross‐species BM stimuli, suggesting a species‐general representation of BM in this brain region, similar to the human results of the hMT+.

### 2.4 No Cluster Functionally Similar to the Human pSTS was Found in the Monkey Brain

Note that in monkeys, we did not find any clusters at a location similar to the human pSTS using univariate analyses. To further investigate the engagement of monkey STS in BM perception, we conducted the following supporting analysis (see Supplemental Material section D for detailed protocols). First, we attempted to identify clusters in the monkey STS based on the multivariate results. Briefly, we performed whole‐brain searchlight analyses for within‐species and cross‐species classifications (Figures S9 and S10, Supporting Information). Based on the results in humans, we conjugated areas that could make successful within‐species classifications and those that could not perform successful cross‐species decoding (Figure S11, Supporting Information). We did find one consistent region anterior to MT in the posterior inferotemporal cortex (TEO) across the three animals, which exhibited similar performance to the pSTS ROI in humans in the multivariate analyses (Figure S13, Supporting Information). However, we did not observe any significant activation differences between the upright and inverted versions across all four types of BM stimuli (Figure S12, Supporting Information).

Second, we conducted functional connectivity analyses similar to those performed in humans to assess the information transmission between MT and TEO in monkeys. The DCM analysis showed that the “bidirectional modulation” had the highest posterior probability for both the macaque and human stimuli (Figure S14A, Supporting Information). Similar to the human results, the driving inputs of the upright BM stimuli were significantly stronger than those of the inverted ones, but only for within‐species BM perception (the macaque BM stimuli in Figure S14B, Supporting Information, *F*_1,51_ = 10.77, *p* = 0.002). This was not the case for cross‐species BM perception (the human BM stimuli in Figure S14B, Supporting Information, *F*_1,56_ = 0.86, *p* = 0.359). However, the modulatory effect of the upright BM was significantly stronger than that of the inverted BM from MT to TEO for both the macaque and human stimuli on the forward connection (Figure S14B, Supporting Information, macaque BM: *F*_1,51_ = 5.73, *p* = 0.020; human BM: *F*_1,56_ = 8.52, *p* = 0.005), but not on the backward connection (Figure S14B, Supporting Information, macaque BM: *F_1_*_,51_ < 0.01, *p* = 0.979; human BM: *F*_1,56_ = 1.45, *p* = 0.233). These results suggest that the monkey TEO does not perform the same function as the human pSTS, indicating that no functionally homogenous brain clusters in monkey STS are completely comparable to the human pSTS in the current study.

Additionally, to further validate the aforementioned assertion, we tried a well‐established cross‐species registration method, specifically the cross‐species functional alignment developed by Xu et al. (2020).^[^ ^34^ ^]^ However, the pSTS region in the macaque brain, identified using cross‐species alignment methods, exhibited significant differences in the representation of BM stimuli compared to the human pSTS (see Supporting Material section E for detailed protocols, Figures S15–S17, Supporting Information).

## 3 Discussion

Our study revealed the brain mechanisms underpinning species‐general and species‐specific BM perception in humans and monkeys. For humans, the hMT+ area responded similarly to both the human and macaque BM stimuli, while the hpSTS area selectively responded to the human BM stimuli. This finding was further supported by increased feedforward connectivity from hMT+ to hpSTS only when processing the human BM stimuli. For monkeys, the MT area could also indiscriminately represent both the human and macaque BM stimuli; however, there were no areas specifically tuned to the macaque BM stimuli. The direct comparison between the findings in humans and monkeys substantiates that both similarity and divergence exist in the brain mechanisms tuned to within‐ and cross‐species BM perception.

Within the third pathway of visual processing, the MT emerges as a primary recipient of visual motion information from the primary visual cortex, prior to the STS during BM perception in both humans and monkeys.^[^ ^29^ ^]^ Previous studies have shown that the hMT+, a region known for its sensitivity to motion, is strongly activated when processing BM information.^[^ ^35^ ^]^ BM stimuli provide a wealth of information, including the shape of the moving subject, the species of the organism, and the intention of the action. Among these, the hMT+ region is predominantly dedicated to deciphering the motion‐related aspects,^[^ ^18^, ^36^ ^]^ particularly the complex coherent motion contained in BM.^[^ ^37^ ^]^ Furthermore, the hMT+ can also be activated by scrambled BM or local feet motion, suggesting that the hMT+ is able to process basic motion aspects in the early stages of BM perception, such as acceleration or opponent motion.^[^ ^21^, ^37^, ^38^ ^]^ Similar to human studies, the monkey MT area is also pivotal for the analysis of visual motion^[^ ^39^ ^]^ and can be significantly activated when monkeys watch either human point‐light displays^[^ ^40^ ^]^ or monkey movement stimuli.^[^ ^27^ ^]^ The view that the MT area functions as an initial motion analysis of the BM stimuli implicitly implies its general ability to encode the BM cues shared across various species,^[^ ^12^, ^38^ ^]^ which has been confirmed by our human and monkey results.

In the third visual pathway, dynamic visual information is transmitted to the STS via the MT.^[^ ^29^ ^]^ Our human results also demonstrated that it is in the hpSTS region that the conspecific BM information is specifically identified. Neurophysiological studies have pinpointed the human pSTS as the region most critically involved in BM perception (for a review, see^[^ ^41^ ^]^; for a meta‐analysis, see^[^ ^19^ ^]^).^[^ ^42^ ^]^ The species‐specific response found in the pSTS is presumably because the conspecific BM inherently carries essential social information for potential communication.^[^ ^43^ ^]^ The human pSTS has been revealed as a hub that integrates relevant social information and processes a plethora of socially significant stimuli,^[^ ^44^ ^]^ including communicative intentions^[^ ^45^ ^]^ and social interaction.^[^ ^31^ ^]^ In line with this explanation, preliminary evidence has shown that the pSTS responds more strongly to human motions compared with animal motions.^[^ ^43^, ^46^, ^47^ ^]^ Our results extended the previous findings by applying a stricter contrast (upright BM versus inverted BM) and revealing increased functional connectivity between hMT+ and pSTS during the perception of conspecific BM. The functional connectivity results clearly portrayed that the cross‐species BM information initially processed in the hMT+ may be toned down during the transition to the hpSTS, particularly due to its limited social relevance for humans.

However, the present study did not find any downstream regions in the STS in monkeys that responded more to upright stimuli than inverted ones, either from the same species or different species. On the contrary, previous electrophysiology and imaging studies discovered that the fundus and upper bank of the posterior and middle STS,^[^ ^27^ ^]^ the mid‐anterior STS region,^[^ ^40^ ^]^ and even the anterior superior temporal polysensory area (the upper bank of the front part of the STS)^[^ ^48^ ^]^ were involved in processing the kinematics of monkey and human BM stimuli, demonstrating that discontinuous portions of the monkey STS can be activated by both within‐ and cross‐species BM stimuli. First, the reason why we did not observe stronger activation to upright BM stimuli relative to inverted BM stimuli in monkey STS may be attributed to the contrast we employed. Different from the contrast between intact and scrambled BM in Krekelberg et al.’s study and the contrast between biological and translational motion in Jastorff et al.’s study, the contrast between upright and inverted BM used in our study selectively captures the kinetics along the direction of gravity.^[^ ^49^ ^]^ Unlike humans, monkeys in the natural environment can not only hang on trees in an upside‐down position but also accumulate rich visual experience from observing other monkeys hanging upside down. Therefore, monkeys may be equally sensitive to upright and inverted BMs compared to humans. Analogously, humans, if exposed to a microgravity environment for a prolonged period, would increase their sensitivity to inverted BM through decreasing activation in the pSTS.^[^ ^33^ ^]^ The natural environment to which monkeys are exposed may shape their STS regions’ responses to inverted BMs.

Second, are there some subregions in the monkey STS that exhibit species‐specific properties when processing BM stimuli? In Jastorff et al.’s investigation, they found the body patches in the STS show stronger activation to conspecifics compared to human action.^[^ ^27^ ^]^ Although the entire monkey STS region is comparably activated by the upright and inverted versions of BM stimuli in the present study, we wondered whether the activation pattern from a certain proportion of it specifically represents within‐species BM rather than cross‐species BM. Indeed, we found one region around TEO, which can represent within‐species BM but cannot represent the shared BM across species, similar to the human pSTS. However, upon examining the functional connectivity between MT and TEO, unlike in humans, we observed very similar results for own‐species and other‐species BM stimuli in monkeys. Hence, the species‐specific BM processing (especially with kinetics along the direction of gravity) exists in monkey STS, but it may not employ the same mechanisms as observed in humans. In light of these results, species‐specific BM perception in monkeys warrants further investigation.

Previous findings have provided ample evidence that the third visual pathway is dedicated to BM processing in both human and non‐human primate brains.^[^ ^27^, ^29^, ^38^ ^]^ Our study advances beyond previous knowledge by illustrating that the neural codes for BM perception in humans and monkeys are not fully duplicated. Specifically, we identified a neural pathway in humans, from hMT+ to hpSTS, that selectively processes conspecific BM, filtering out BM from other species. Conversely, in monkeys, BM from all species is generally processed in the MT, without a similar species‐specific representation in the STS.

This result agrees with the study by Joly et al. (2012), who conducted a functional MRI study in humans and monkeys to investigate conspecific vocalizations in both species. Results showed that monkeys and humans exhibited similar fMRI activity maps in the primary auditory cortex. In contrast to monkeys, humans showed a clear conspecific preference in the STG/STS. Thus, they proposed that the evolution of the language faculty in humans is primarily reflected in the STS region.^[^ ^50^ ^]^ Similar results are found for dynamic face processing. A comparative fMRI study revealed that the specialization of the STS for dynamic facial expressions is both stronger and more specific in humans than in monkeys, indicating that the human STS may possess unique properties that enhance its ability to process social cues.^[^ ^51^ ^]^ Furthermore, it has been demonstrated that the STS forms a specialized hub for processing social information in humans (including faces, human bodies, biological motion, goal‐oriented actions, social interaction, etc.),^[^ ^44^ ^]^ but there are few related findings in monkeys. The current results align with the evolutionary perspective of brain development, indicating that the brain undergoes a continuous process of evolution, with the downstream (high‐level brain regions) being especially driven toward differentiation and specialization.^[^ ^52^ ^]^ This striking difference may reflect the distinct evolutionary trajectories of each species after diverging from their common ancestor.

The study examined species‐general and species‐specific BM perception by comparing the upright and inverted versions of human and macaque point‐light BM stimuli. While these stimuli allowed control over low‐level visual features, they lacked the vividness of videos, which might diminish social relevance and result in weaker hpSTS activation compared to hMT+. Perhaps other actions besides walking would convey much clearer social interactive information.^[^ ^53^ ^]^ In addition, the increased familiarity with our own species compared to other species might complicate ascertaining whether the selectivity of the hpSTS for human BM is entirely driven by species‐specific behaviors. It should be noted that familiarity and species‐specificity are two facets of the same coin. They cannot be completely separated from each other as we are naturally familiar with our own species. To thoroughly rule out the familiarity effect in BM perception, future research might need to investigate infants who have had limited visual exposure, thus minimizing the influence of prior experience. Another concern related to the BM walking stimuli is that these stimuli may be too abstract and simplified for monkeys to correctly recognize their conspecies. We cannot exclude the possibility that some regions in the monkey STS would respond to the same‐species BM when more details other than walking kinetics are provided. Future research should use more ecological stimuli (e.g., videos) and aim for larger sample sizes to enhance statistical power.

## 4 Conclusion

The present study innovatively adopts a cross‐species comparison approach to delve into the neural substrates involved in both within‐species and cross‐species BM perception, as well as its divergence across species. These findings not only enrich our understanding of the evolutionary aspects of the third visual pathway, but also pave the way for future research, particularly in considering species differences in processing biosocial information.

## 5 Experimental Section

### Humans

Twenty‐one healthy adults (11 males, mean age 25 ± 3 years) were enrolled in the fMRI experiment. All participants had normal or corrected‐to‐normal vision and provided written informed consent before the formal experiment. The current study was conducted in accordance with the Declaration of Helsinki and was approved by the institutional review board of the Institute of Psychology, Chinese Academy of Sciences (Protocol Number: H21058). Two participants were removed because their ROIs (regions of interest) could not be reliably found under the predefined threshold, and one participant was removed due to excessive head movements (>2 mm). Thus, eighteen participants were included for further analysis.

### Monkeys

Three male macaque monkeys (monkeys Q, K, and M; Macaca mulatta; 10–11 y old; 7.0 – 10.5 kg) were used. They were acquired from the same primate breeding facility in China, where they had social group histories as well as group‐housing experience until their transfer to the Institute of Biophysics (IBP), CAS, for quarantine at the age of ≈4y. Animals used in this study had been housed at IBP for 6–7 y before this experiment. All experimental procedures complied with the US National Institutes of Health Guide for the Care and Use of Laboratory Animals and were also approved by the Institutional Animal Care and Use Committee of IBP (Protocol Number: IBP‐NHP‐003). Each monkey was surgically implanted with a magnetic resonance (MR)‐compatible head post under sterile conditions, using isoflurane anesthesia. After recovery, subjects were trained to sit in a plastic restraint chair and fixate on a central target for long durations with their heads fixed, facing a screen on which visual stimuli were presented.^[^ ^54^, ^55^, ^56^ ^]^

### Stimuli

For both the human and monkey fMRI experiments, we used the same set of human and macaque BM stimuli. Human point‐light BM stimuli were adopted from motion capture data,^[^ ^57^ ^]^ which consisted of 15 dots located at the major body joints from the head, shoulders, elbows, wrists, hips, knees, and ankles of human actors (see Figure 1A). We then randomly presented six viewpoints of the point‐light walkers, each representing a rotation of the direction in which the walkers were moving, uniformly distributed from 90° leftwards to 90° rightwards relative to the horizontal axis of the screen, to avoid neural adaptation in the fMRI experiment. Macaque point‐light BM stimuli were converted from three full‐body macaque video clips depicting their walking movement from the side. It was first manually tracked the motion sequences of 20 major joints in the four legs (3 × 4), head (3), back (3), and tail (2) of monkeys frame by frame (see Figure 1A). To keep the macaques walking in place, the positions of the point‐light BM stimuli was centralized to cancel the translation. Their moving trajectories were further smoothed by realigning the position of each joint at each frame to a virtual position lying linearly between its preceding and following positions, while keeping the distances from this position to its preceding and next positions in the same ratio with the distances calculated using the original positions. This algorithm could effectively eliminate the irregular moving jitters without affecting the core kinetics of biological agents. The three‐macaque point‐light BM stimuli walked toward the left or right (i.e., six distinctive stimuli comparable to the human BM stimuli).

Additionally, scrambled counterparts for each intact stimulus were generated by randomizing the initial position of each point within the same region. Such manipulation keeps their local motion trajectories intact but disrupts the global configuration. As controls, inverted BM stimuli were created by vertically mirror‐flipping the four upright BM sequences (i.e., intact human, scrambled human, intact macaque, and scrambled macaque).

### fMRI Acquisition

*Humans*: Functional and anatomical MRI scanning was carried out in the Beijing MRI Center for Brain Research (BMCBR) at a 3‐Tesla Siemens Prisma MRI scanner with a 20‐channel head coil. For anatomical scans, a high‐resolution T1‐weighted structural image was obtained with the following parameters: voxel size: 1 mm isotropic; repetition time (TR): 2600 ms; echo time (TE): 3.02 ms; flip angle (FA) = 8°; field of view (FOV): 240 × 240 mm; Functional images were acquired with the following parameters: T2*‐weighted gradient – multiband accelerated echo‐planar imaging (EPI) sequences; Imaging parameters were as follows: voxel size: 2 mm isotropic TR:2000 ms; TE:30 ms; FA: 70 °; FOV: 192 × 192m.

*Monkeys*: Functional and anatomical MRI scanning was performed with the same equipment as humans with an 8‐channel head coil. Before each scanning session, an exogenous contrast agent [monocrystalline iron oxide nano colloid (MION)] was injected into the femoral or external saphenous vein (8 mg kg^−1^) to increase the contrast/noise ratio and to optimize the localization of fMRI signals.^[^ ^58^ ^]^ Forty‐eight 1.5 mm coronal slices (no gap) were acquired using single‐shot interleaved gradient‐recalled echo planar imaging. Imaging parameters were as follows: voxel size: 1.5 mm isotropic; TR: 2.5 s; TE: 17 ms; FA: 90°, FOV: 129 × 129 mm. A low‐resolution T2 anatomical scan was also acquired in each session to serve as an anatomical reference (0.625 mm × 0.625 mm × 1.5 mm; TE: 101 ms; TR: 11.2 s; flip angle: 126°). To facilitate cortical surface alignment and the following local targeting, we also acquired high‐resolution T1‐weighted whole‐brain anatomical scans in separate sessions. Imaging parameters were as follows: voxel size: 0.5 mm isotropic; TR: 2.2 s; TE: 2.84 ms; flip angle: 8°.

### Human fMRI Experiments

*Main experiment*: The main fMRI experiment adopted a within‐subjects block design. Participants were scanned in four sessions: scrambled human BM, intact human BM, scrambled macaque BM, and intact macaque BM. The session order was counterbalanced across participants, but scans of scrambled stimuli were always before scans of intact stimuli to avoid recognizing scrambled stimuli once intact stimuli were shown. Each session had two runs. In each run, eight blocks of upright and inverted BM displays were presented in a counterbalanced order. In sum, across the four conditions—scrambled human BM, intact human BM, scrambled macaque BM, and intact macaque BM—each consisted of 32 blocks, equally divided into 16 upright and 16 inverted configurations. Each block lasted 12 s, followed by a 6s interval. During each block, taking intact human BM as an example, participants viewed six exemplars of random BM stimuli from different viewpoints, which were consecutively presented for 2s each (see Figure 1C). A red fixation (0.5° × 0.5°) appeared in the center against the gray screen during the whole run. Human point‐light BM and macaque point‐light BM stimuli are subtended ≈2.3°× 6.6°and 5.3° × 3.6° in visual angle, respectively. To further avoid neural adaptation, the BM stimuli to float with a random offset of fixation within an area of 1° was allowed. Participants were required to maintain their attention on the central fixation and to press a response button when the fixation changed its color to a light red. In the scanner, all stimuli were presented using MATLAB (MathWorks, Inc.) with the Psychophysics toolbox extension.^[^ ^59^ ^]^ Participants viewed the screen (60 Hz frame rate) through a mirror mounted on the head coil.

*Localizer*: To eliminate data circularity issues,^[^ ^60^ ^]^ participants completed three additional localizer scans after the main experiment. According to the previous study,^[^ ^61^, ^62^ ^]^ two functional localizer tasks were performed to define the human and macaque BM‐sensitive STS regions (abbreviated as hpSTS‐human and hpSTS‐macaque), respectively, by contrasting the upright intact human/macaque BM with its inverted scrambled counterpart. In the subsequent ROI, same species MVPA, and DCM analysis, we used the hpSTS‐human ROI for human BM condition analysis, and the hpSTS‐macaque ROI for the monkey BM condition. In addition, the hpSTS‐combined ROI for the cross‐species MVPA was defined, using the contrast of human + macaque upright intact BM > human + macaque inverted scrambled BM (MNI coordinates: right hpSTS‐combined, [x y z] = 50, ‐38, 11; left hpSTS‐combined, [x y z] = ‐54, ‐44, 11), which could ensure a more consistent and unbiased definition of pSTS across both species. All procedures for these tasks were similar to those of the main experiment. In each task, there were 12 upright intact BM blocks and 12 inverted scrambled BM blocks across two runs. Each block lasted 12 s, followed by a 6s interval. During each block, six exemplars of human BM from different viewpoints were consecutively presented for 2 s each. A red fixation appeared in the center against the gray screen during the whole run. Participants were instructed to detect the fixation color change. The third localizer task was used to define the hMT+. The design of this task was also identical to the main experiment except that the BM stimuli were replaced by moving optic flow (expanding and contracting) and static dots, which were presented within a circular region of radius 3.2°.^[^ ^25^ ^]^ Similarly, stimuli were shown in 24 blocks in 2 runs (each condition contained 12 blocks) in a counterbalanced order. Each block lasted 12 s and six exemplars of the random stimulus were displayed for 2 s each. Participants were again instructed to detect the fixation color change.

### Monkey fMRI Experiments

*Main experiment*: The main fMRI experiment shared almost the same design and procedure as the human experiment. Here, the critical and dissimilar parts were briefly introduced. As human participants, three monkeys also viewed four types of BM stimuli: scrambled human BM, intact human BM, scrambled macaque BM, and intact macaque BM. In each session, 14–21 runs were collected from each animal, and the detailed information was presented in Table S7 (Supporting Information). In each run, the upright and inverted BM stimuli were displayed in 4 blocks, respectively, in a counterbalanced order. Each block had a duration of 30 s, followed by a 15 s fixation interval. Six BM stimuli with different viewpoints were consecutively presented for 2.5 s and repeated twice during each block. The human BM and macaque BM stimuli were subtended ≈3.6°×10.3° and 9.1° × 6.2° in visual angle, respectively.

Monkeys were required to maintain fixation on a central square on the screen during the whole experiment and receive a liquid reward. The monkeys’ eye position was tracked using an infrared pupil tracking system (ISCAN, Inc). The frequency of reward would be increased as the duration of fixation increased.^[^ ^54^, ^55^, ^56^ ^]^ The fMRI data enrolled in subsequent analysis only came from those blocks/runs with qualified fixation percentage (Monkey Q, upright: 89.90 ± 0.58%, inverted: 89.74 ± 0.62%; Monkey K, upright: 89.00 ± 0.57%, inverted: 88.38 ± 0.61%; Monkey M, upright: 90.54 ± 0.58%, inverted: 90.69 ± 0.62%).

*Localizer*: Monkeys performed three localizer tasks as humans. The BM stimuli used to define the ROIs sensitive to human and macaque BM stimuli and the moving dots used to define the ROI sensitive to pure physical motion were identical to the localizer tasks of humans. All other parameters were the same as those in the main fMRI experiment of monkeys. The run numbers collected in the three localizer tasks were presented in Table S7 (Supporting Information) as well.

### Human Data Analysis

*Preprocessing*: Preprocessing was implemented with SPM12 (http://www.fil.ion.ucl.ac.uk/spm), and ROI analysis was performed with MarsBar (http://marsbar.sourceforge.net). The imaging data preprocessing involved re‐slicing, co‐registration, segmentation, normalization, and smoothing. Specifically, EPI volumes were realigned using the first volume of each run as a reference. Then, the structural image was registered to the reference image and segmented into gray matter, white matter, and CSF in each individual space. The segment parameters were further used to normalize each individual's functional images onto the SPM MNI152 template. After normalization, data were spatially smoothed with an FWHM, 4 mm Gaussian kernel.

### Whole‐Brain Analysis

All data were fitted through a general linear model (GLM). For each type of BM stimuli (i.e., scrambled human, intact human, scrambled macaque, intact macaque), the GLM analyses included two regressors of interest (upright and inverted versions) and six motion regressors of no interest (three translation parameters and three rotation parameters). All regressors were convolved with the hemodynamic response function. Two contrasts, upright > inverted and inverted > upright, were computed for each participant in a first‐level t‐test. To show group‐level statistical maps across subjects, contrast images of all the participants were included in a second‐level group analysis, with an FDR‐corrected threshold of *p* < 0.05 at the cluster level (*p* < 0.001 uncorrected at the voxel level) and an extent threshold of 10 voxels.

### ROI Definition

According to the previous study, hpSTS‐human ROI was selected in individual normalized functional data sets, with the selection of the individual local maxima (upright intact human BM > inverted scrambled human BM (*p* < 0.05, uncorrected) located closest to the superior temporal cortex region (MNI space). Each ROI was defined as a sphere with a 6 mm radius, centered on the identified local maximum. In the same vein, we identified the hpSTS‐macaque ROI sensitive to macaque BM with the contrast of upright intact macaque BM > inverted scrambled macaque BM (*p* < 0.05, uncorrected) and the hMT+ with the contrast of moving dots > static dots (*p* < 0.005, uncorrected). The ROIs were also applied in the subsequent PPI and DCM analysis. Their mean MNI coordinates for all participants were described in Table S1 (Supporting Information), and the locations of these ROIs for each participant were displayed in Figure S1 (Supporting Information).

### MVPA Analysis

To further examine which brain regions encode the upright and inverted BM stimuli, we conducted a multivariate pattern analysis (MVPA) implemented in the COSMOMVPA toolbox.^[^ ^63^ ^]^ In this analysis, the functional images were spatially smoothed by 2 mm, but they were not normalized. ROIs were created in individual native spaces. We trained the support vector machine (SVM) classifier ^[^ ^64^ ^]^ to distinguish between brain activation patterns (beta values) of upright and inverted BM stimuli in a block‐based fashion. We trained the classifier on data from 31 out of 32 blocks (leaving one out). We assessed the accuracy of the classifier by testing it on the upright/inverted BM stimuli in the held‐out block. That was iterated 32 times, and the classification accuracy was averaged for each subject and ROI.

To assess whether the representation of BM is generalized across species, a cross‐classification MVPA was conducted. The classifier was trained to differentiate between brain activation patterns (beta values) for the upright and inverted versions of the intact human BM stimuli but tested on its accuracy at classifying the counterparts of the intact macaque BM stimuli and vice versa. The same approach was repeated for the scrambled BM stimuli. For each ROI, individual classification accuracies were subsequently compared against the chance level with one‐sample t‐tests (α = 0.05, two‐tailed, Bonferroni corrected for 4 comparisons, 2 hemispheres × 2 ROIs). Significant cross‐classification accuracy demonstrates shared representation underlying human and macaque BM perception.

### PPI and DCM Analysis

To investigate the functional coupling among brain regions related to BM perception, an exploratory PPI analysis ^[^ ^65^ ^]^ was conducted. The previously defined bilateral hMT+ ROIs was selected as a seed region and the first eigenvariate time series from them as the physiological term was extracted. The interaction term of PPI analysis was calculated by multiplying the hMT+ ROI activity and psychological variable of interest (upright versus inverted).^[^ ^66^ ^]^ Afterward, the convolved regressor for the interaction term, the physiological variable (i.e., the hMT+ activity), and the psychological variable were entered into a first‐level GLM analysis, where the six head movement parameters were treated as nuisance regressors. The interaction terms for individuals’ contrast images were further estimated to create group‐level maps (*p* < 0.05 after FWE correction).

Given that PPI analysis does not reveal the direction of the functional connectivity between brain regions, a DCM analysis was conducted in the DCM tool of SPM12. Based on previous studies and the key implication of the PPI results, the analysis focused on the effective connectivity between hMT+ and hpSTS regions when human and macaque BMs were displayed. It was worth noting that in the DCM analysis, the hpSTS‐human ROI was used for human BM display, while the hpSTS‐macaque ROI was used for macaque BM display. For each participant, the same ROIs in the left and right hemispheres were combined for further analysis. Then, the activity time series for each volume of interest was extracted by computing the first eigenvector of all its voxels.

The DCM models were constructed by first assuming that the driving visual input (here, both the upright and inverted BM stimuli) through hMT+ entered the system (C‐matrix). Second, it was assumed that the intrinsic connection between the hMT+ and the hpSTS was bidirectional (hMT+ ↔ hpSTS; A‐matrix). To reveal how BM perception modulates the endogenous connectivity between hMT+ and hpSTS, the modulatory effects of BM perception (upright versus inverted) on the intrinsic connections between MT and hpSTS (B‐matrix) were systematically varied in 4 models: 1) the “no modulation” mode; 2) the “forward modulation” mode, in which the connection from hMT+ to hpSTS is unidirectionally modulated by BM perception; 3) the “backward modulation” mode, in which the connection from hpSTS to hMT+ is unidirectionally modulated by BM perception; 4) the “bidirectional modulation” mode, in which both feedforward and feedback connections between hMT+ and hpSTS are modulated by BM perception. For illustrative purposes, all the hypothesized models were drawn in Figure 3A. Then, we conducted the model comparison using a fixed‐effects Bayesian model selection (BMS), and the most appropriate model was assessed by exceedance probability.^[^ ^67^ ^]^ The parameter values of the winning model relating to the modulation effect were assessed using paired t‐tests at the group level.

### Monkey Data Analysis

*Preprocessing*: Image preprocessing was conducted with AFNI.^[^ ^68^ ^]^ Briefly, the EPI volumes were time‐corrected and realigned to the base volume with minimum outliers. Then, outliers of EPI images were eliminated with the 3dDespike function. EPI volumes were next smoothed with a 2 mm‐full‐width half‐maximum Gaussian kernel and normalized to the mean signal value within each run. The EPI images were linearly registered to the low‐resolution T2 images, which were rigidly registered to the high‐resolution T1 images, and these were then nonlinearly registered to the NIMH Macaque Template (NMT v2) space.^[^ ^69^ ^]^ Consistent with previous fMRI studies on awake monkeys,^[^ ^61^, ^70^, ^71^, ^72^ ^]^ our analyses were conducted on a volumetric basis, which was appropriate given the spatial resolution used (i.e., 1.5 mm isotropic, typical for monkey data from a 3T scanner). Tissue segmentation was rarely, if ever, applied in such studies for analyzing functional activations, and consequently, tissue segmentation was not employed in the current study.

### Whole‐Brain Analysis

After preprocessing, a GLM was fitted to estimate and compare the neural activities between the upright and inverted versions for each type of BM stimuli (i.e., scrambled/intact human, scrambled/intact macaque). In addition to the above regressor of interest, each GLM model included a hemodynamic response function with the MION response curve and regressors with no interests (baseline, movement parameters from realignment corrections, and signal drifts). For each type of BM stimuli, the contrast map (upright versus inverted) was computed for each monkey with an FDR‐corrected threshold of *p* < 0.05 at the cluster level (*p* < 0.001 uncorrected at the voxel level and an extent threshold of 5 voxels). Finally, a cluster was determined to be a ROI selectively responding to BM stimuli if it was found in similar anatomical locations across all three monkeys. All fMRI signals throughout the study were inverted so that an increase in signal intensity indicates an increase in activation.^[^ ^73^ ^]^

### ROI Definition

The monkey ROI analysis was performed using similar contrasts as humans (e.g., upright intact BM > inverted scrambled BM or moving dots > static dots) and adjusted for the following reasons. First, as almost all areas along the monkey STS exhibited significant differences in the MT functional localizer (uncorrected *p* < 0.001, FDR corrected *p* < 0.05), the top 60 voxels that fell within the MT anatomical area (dilated by one voxel using AFNI 3dmask_tool) at each hemisphere was selected using the D99 macaque brain atlas.^[^ ^74^ ^]^ Given its relative stability in macaques,^[^ ^75^, ^76^, ^77^ ^]^ the cluster was named the MT as a comparable ROI to human hMT+.^[^ ^27^, ^78^, ^79^, ^80^ ^]^ The location of the anatomical MT area has been demonstrated considerable consistency compared to other atlases, such as the Paxinos macaque atlas (Figure S4, Supporting Information) published by the Center for in vivo Microscopy (CIVM).^[^ ^81^ ^]^ The voxels comprising the MT ROI were contiguous. Second, in both the human and monkey BM localizers, we were not able to find a functionally homogenous cluster in the monkeys comparable to the pSTS in humans, which means no clusters in the STS responded more strongly to the upright intact BM stimuli than the inverted scrambled BM stimuli (except those areas close to and overlapped with the MT ROI, see Figure S4, Supporting Information). Therefore, only the MT ROI was localized for the monkeys, with detailed information in Table S8 and Figure S4 (Supporting Information).

### MVPA Analysis

The MVPA was also conducted using the COSMOMVPA toolbox. For each monkey, an SVM classifier was trained using data from the predefined ROI to distinguish between the upright and inverted versions for each type of BM stimuli in a run‐based fashion. The leave‐one‐run‐out cross‐validation was used to get the classification accuracy,^[^ ^82^ ^]^ and this process was iterated N times (N was the total number of runs for each monkey). Following the human analysis, such manipulation was conducted within and cross species.

### Statistical Analysis

*Human data analysis*: For the ROI analysis, the beta values of the BOLD signal within all the ROIs for each participant was extracted. These values were further analyzed by a 2 (hemisphere: left and right) × 2 (orientation: upright and inverted) repeated‐measures ANOVA for each type of BM stimuli, and a post‐hoc analysis was performed to further elucidate the specific differences between upright and inverted conditions.

For the MVPA analysis, the accuracies were entered into a one‐sample t‐test and were compared to a 50% chance level (α = 0.05, two‐tailed, Bonferroni corrected for 4 comparisons, 2 hemispheres × 2 ROIs). The above analysis was separately conducted for scrambled human, intact human, intact macaque, and scrambled macaque BM stimuli.

### Monkey Data Analysis

For the ROI analysis, to compare the beta values of the MION signals from the upright and inverted version, a 2 (hemisphere: left; right) × 2 (orientation: upright; inverted) LMM with runs, sessions, and monkeys as random factors was run on the MT ROI ^[^ ^56^, ^72^, ^83^ ^]^ for each type of BM stimuli respectively (i.e., scrambled/intact human, scrambled/intact macaque). All the models were fitted in the R programming version 3.6.3 (nlme and emmeans packages).

For the MVPA analysis, given the small sample size in the monkey experiments, we used a binomial test to evaluate the statistical significance of classification accuracies for both within‐ and cross‐species decoding.^[^ ^82^ ^]^ We enrolled all runs from 3 monkeys for the group analysis (Bonferroni corrected for 2 comparisons, 2 hemispheres × 1 ROI) and all these statistical tests were performed in the R programming version 3.6.3 (the stats package).

## Conflict of Interest

The authors declare no conflict of interest.

## Author Contributions

Y.C. and Y.X. contributed equally to this work. X.Y., N.L and Y.J. performed conceptualization; Y.C., Y.X., X.L. and N.L. performed methodology; Y.C., Y.X., T.Y and X.M. performed investigation; Y.C., and Y.X. performed visualization; N.L. and Y.J. performed supervision; Y.C., Y.X and X.L. wrote the original draft, X.Y., N.L. and Y.J. wrote, reviewed and edited.

## Supporting information



Supporting Information

## Data Availability

The data that support the findings of this study are openly available in Psychological Science Bank at https://doi.org/10.57760/sciencedb.psych.00176, reference number 176.
